# Topical Peptide Technology and the Scarring Spectrum: Linking Mechanistic Pathways to Internal Scarring (Fibrous Banding) and External Scarring (Incisional) and Outcomes in Postsurgical Healing

**DOI:** 10.1093/asjof/ojaf169

**Published:** 2025-12-18

**Authors:** Alan D Widgerow, Laurie A Casas, Brannon Claytor, Kamakshi R Zeidler, Faiza Shafiq

## Abstract

Surgery results in external scars and internal scars (subcutaneous fibrous banding). Although external scarring is well understood, internal fibrous banding is less studied, despite its impact on comfort, mobility, and recovery in surgical patients. Alastin ReFORM & RePAIR COMPLEX with TriHex Technology (R&R) is a peptide-based formula that remodels the extracellular matrix (ECM). It has been tested in multiple clinical trials, showing it stimulates autophagy and macrophage repolarization. The aim was to outline the mechanistic and clinical evidence related to both internal and external scarring. They conducted a secondary analysis of 3 split-body clinical trials, with proposed mechanistic explanations extrapolated. Endpoints assessed included patient-reported outcome measures, blinded investigator ratings, ultrasound, histology, and photographic scar evaluations. Cellular secretome, interleukin-6 (IL-6) modulation, and autophagic clearance of lipid droplets and ECM remodeling provide a mechanistic explanation for the observed effects. In body contouring procedures, R&R significantly accelerated the resolution fibrous banding compared with comparators in split-body studies. Ultrasound confirmation and histology showed reconstituted ECM on the R&R-treated side. External scar assessment revealed improved subjective scar quality over time. These outcomes are mechanistically linked to the autophagic clearance of lipid droplets, ECM remodeling, and macrophage polarization, which are influenced by IL-6 and modulate the transition from inflammation to regeneration. R&R influences the spectrum of surgical scarring, affecting both internal and external scars. The scar spectrum broadens traditional views of postoperative healing, emphasizing internal fibrous banding as a measurable outcome and positioning a topical formulation as a credible additional tool in perisurgical care.

**Level of Evidence**: 2 (Therapeutic) 
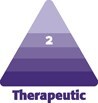

Assessment of postsurgical healing has mainly focused on external scars. Outcomes evaluate the appearance of the incision line using established scar assessment scales such as the Vancouver Scar Scale or the Patient and Observer Scar Assessment Scale, examining factors like thickening, erythema, pigmentation, and overall appearance.^1^ Of course, these are important criteria for outcome assessment, but this narrow focus overlooks another vital aspect of postsurgical healing: internal scarring. This presents as subcutaneous fibrous banding, induration, and tissue tightening.

Body contouring surgeries, including procedures like liposuction, abdominoplasty, and breast surgery, all involve an internal fibrotic process. Patients often describe the induration as hardness or lumps, and the tight bands can limit mobility and cause discomfort. This is not a new subjective phenomenon, but it has rarely been measured as an objective endpoint in postsurgical assessments. Persistent internal fibrous banding and inflammation lead to discomfort, prolonged swelling, and restricted movement. However, it has been accepted as a normal part of recovery, with only a few treatments (such as lymphatic massage) addressing it.

ReFORM & RePAIR (R&R) COMPLEX with TriHex Technology (Alastin, a Galderma Company, Carlsbad, CA), a topical formulation designed to remodel and recycle the extracellular matrix (ECM), has been tested in multiple clinical trials.^2-5^ In split-body studies, these trials consistently showed reductions in edema, induration, bruising, and fibrous banding compared with control moisturizers.^4-6^ A comprehensive evaluation of patient-reported outcome measures (PROMs), ultrasound imaging, and histological and gene profile analysis of biopsies has reinforced these clinical findings. They demonstrate that the benefits of R&R extend beyond superficial skin healing to deeper cellular and subcutaneous layers, with a direct impact on postsurgical recovery.^4-6^

A focus on mechanistic insights provides a foundation for explaining these observed dual benefits. Adipocytolysis, the breakdown of fat cells and release of lipid droplets, directly contributes to increased levels of interleukin-6 (IL-6). This inflammatory cytokine modulates and regulates the “on-off” switch for inflammation and potential regeneration that follows.^7^ R&R, through its stimulation of autophagy and lipophagy, promotes early dissolution and absorption of lipid droplets, reducing IL-6-mediated inflammation and triggering macrophage polarization toward the M2 phenotype, which supports regenerative ECM remodeling and healing. This remodeling of the ECM and the creation of a regenerative environment are equally important for external incisional scarring outcomes, where control of inflammation, hydration, and collagen maturation is critical to achieving a favorable scar.^8^ In addition R&R affects Suppressor of mother against decapentaplegic (SMAD) pathways and transforming growth factor (TGFb) analogues that aid in preventing hypertrophic scarring. This offers a unifying strategy for analyzing postsurgical scarring outcomes.

This paper compiles the findings of these trials, integrating the clinical and mechanistic results into a proposed concept of a surgical scarring spectrum that includes both internal and external scarring, which can be influenced and interconnected through ECM dysregulation and modulation. By grouping these healing parameters under the umbrella of the postsurgical scarring spectrum, we expand the definition of postoperative healing and introduce an additional measurable endpoint related to internal scarring. Furthermore, the idea of a topical formulation for preconditioning and perioperative management of the scarring spectrum shifts the approach toward optimizing postsurgical recovery. The aim is to evaluate surgical scarring outcomes, considering both internal and external scarring and to frame this as a unified concept of the surgical “scarring spectrum.”

## METHODS

### Study Design

This publication presents a secondary synthesis and scientific narrative based on published clinical trials, along with mechanistic explanations of the results observed with the use of R&R in surgical patients. Data sources: 4 prospective, controlled clinical trials and 1 pilot study were included: (1) Pilot study—Kubler 2020, observational and PROM study treatment group vs control cohort; (2) Claytor 2021—split-body medial thigh liposuction, histology, gene analysis, PROM evaluation; (3) Casas 2021—body contouring procedures, split-body investigation with ultrasound imaging, investigator assessments (blinded), and PROMs; (4) Multicenter Surgery Trial 2022—randomized, double-blind, split-body, multicenter body contour surgery trial using photography, biopsies, ultrasound imaging, and blinded investigator and participant PROM assessments.^2-5^ The mechanistic review contextualizing the clinical outcomes was based on Widgerow 2021.^7^ Overall, this amounted to 60 patients analyzed with statistical analytic methods described. Outcomes examined included internal scarring such as induration and fibrous banding from the PROM perspective, ultrasound evidence of fibrous tissue, histology of ECM, and gene expression markers. External scarring was characterized by the appearance of the incision line, pigmentation, thickening, and photographic documentation. Mechanistic factors involved IL-6 signaling, autophagy, macrophage polarization, elastogenesis, and ECM remodeling.

No scale exists to objectively measure fibrous banding as an outcome, because it has not been previously explored. We initially used a fibrometer in 1 trial but found it too user-dependent and unreliable. We then decided to combine ultrasound views with clinical examinations for the best assessment. We attempted to establish a baseline quantitative analysis, but ultimately, it was about determining how clear the subcutaneous tissue was from ‘debris’ during recovery. Although not consistent, this method proved to be better. In the end, the clinical examination was the most effective, especially since we were comparing 2 sides—this included pain on pressure, limitation of movement, and any induration of the skin by feel. These assessments were used to evaluate several parameters. At each postoperative visit, the investigator or designee completed an assessment of the right and left sides using a 5-point scale (0—none; 1—barely perceptible, visually or palpably; 2—mild; 3—moderate; and 4—severe) for symptoms, such as ecchymosis, swelling, skin discoloration, induration, and subcutaneous fibrous banding. Fibrous banding was assessed with the same scale, focusing on pain on pressure, limitation of movement, and/or induration—graded based on the significance of these findings. The visual analog scale 0-10 pain scale was administered in person to the participant at each visit.

### Integration of Data

PROMS, ultrasound, histology, and mechanistic findings were extracted and organized into internal and external scar categories and compared across the studies. This was based on descriptive rather than meta-analytic evaluation because of a wide range of heterogeneous procedures. Mechanistically, the cellular pathways were linked to recovery patterns.

The scarring spectrum was defined as the process of healing surgical wounds involving internal scarring (fibrous banding) and external scarring (incisional scar), both linked by ECM mechanisms influenced by R&R.

## RESULTS

### Internal Scarring (Subcutaneous Fibrous Banding)

**Figure 1.**
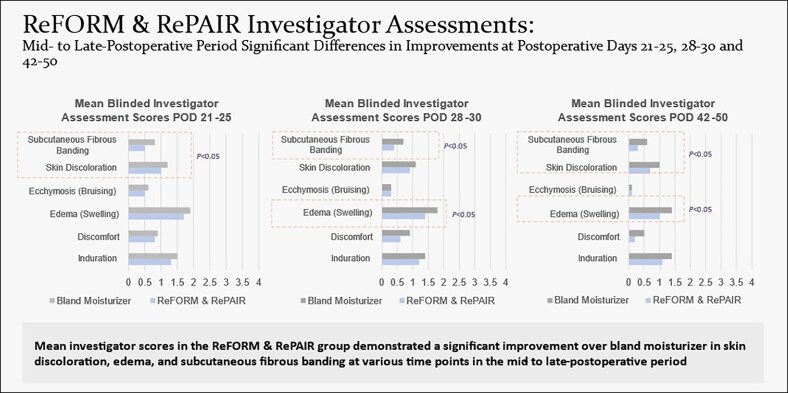
Full statistical analysis across multiple measured parameters in the 3 to 6 week recovery period with edema and fibrous banding showing improved resolution on the R&R side.

**Figure 2.**
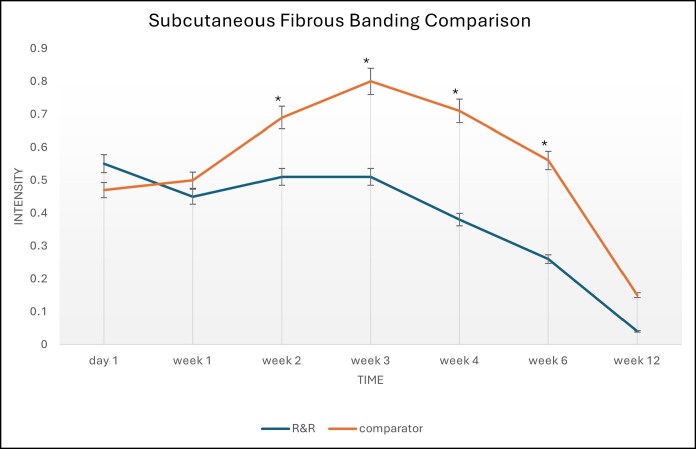
Graph showing subcutaneous fibrous banding changes over 12 weeks between R&R and comparator, with less fibrous banding observed in R&R vs comparator.

In the multicenter trial, 29 females, aged between 31 and 69 years with a mean age of 48 years, completed the study. Participants were followed for 12 weeks postoperatively and attended 7 postoperative visits. The study evaluated 38 surgical body contouring procedures, with participants randomly assigned to R&R on 1 side and a bland moisturizer on the other with both products packaged in an identical fashion. For each procedure and each time point, the score difference between the side treated with the bland moisturizer and the side treated with R&R was calculated. To determine whether there was a significant difference between the 2 sides, both a parametric test (paired *t* test) and a nonparametric test (signed rank test) were used. Blinded investigators found a significant statistical improvement on the R&R side compared with the bland moisturizer. Notably, at no measurement point did the comparator show a statistical advantage over R&R from the blinded investigator's perspective. In this larger, multi-site trial with more time points, the most critical sign of internal scarring—fibrous banding—showed significant differences at 3, 4, 6, and 12 weeks. Additionally, induration and edema also demonstrated significant differences after the 2-week mark.^5^ Statistical analysis revealed the following (Figures 1, 2):

- Ecchymosis at postoperative day (POD) 10-14 (difference = 0.2, standard deviation [SD] = 0.6, *P* = .0296)
- Skin discoloration at POD 21-25 (difference = 0.2, SD = 0.5, *P* = .0141)
- Subcutaneous fibrous banding at POD 21-25 (difference = 0.3, SD = 0.5, *P* = .0025)
- Subcutaneous fibrous banding at POD 28-30 (difference = 0.3, SD = 0.6, *P* = .0057)
- Edema at POD 28-30 (difference = 0.4, SD = 0.7, *P* = .0035)
- Skin discoloration at POD 42-50 (difference = 0.3, SD = 0.7, *P* = .0096)
- Edema at POD 42-50 (difference = 0.3, SD = 0.7, *P* = .0056)
- Subcutaneous fibrous banding at POD 42-50 (difference = 0.4, SD = 0.9, *P* = .00213)

Participants also confirmed the findings of improvement in skin discoloration and fibrous banding over very similar time periods. Additionally, 7 patients broke code, all on the R&R side, which is probably the strongest reflection of patient preference and the efficacy of the product.

In the Kubler trial—investigator and PROM results were recorded as “less postprocedural swelling, less pain using the Visual Analog Pain Scale, and less palpable discomfort in the patients utilizing the topical body treatment (Cohort 1) in all procedural categories when compared with the control group (Cohort 2) that did not use the topical product post procedure. By 6 weeks, rapid resolution of the soft tissue induration and fibrosis was noted in the treated subjects, unlike the control group who experienced persistent palpable and visible soft tissue changes out to 4 months. No adverse events were reported from utilizing the topical body treatment.”

**Figure 3.**
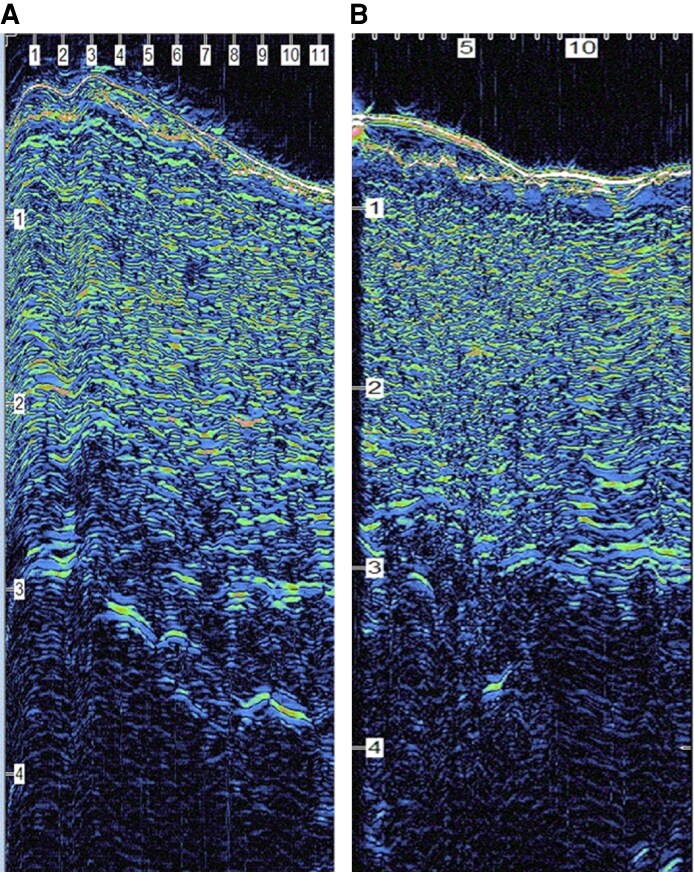
Ultrasound image of an abdominoplasty 6 weeks postsurgery (A) with bland moisturizer and (B) with R&R—showing decreased fibrous banding in B (less debris within subcutaneous tissue and better separation between dermis and subcutaneous tissue). Subtle but noticeable changes typical of ultrasound results observed.

**Figure 4.**
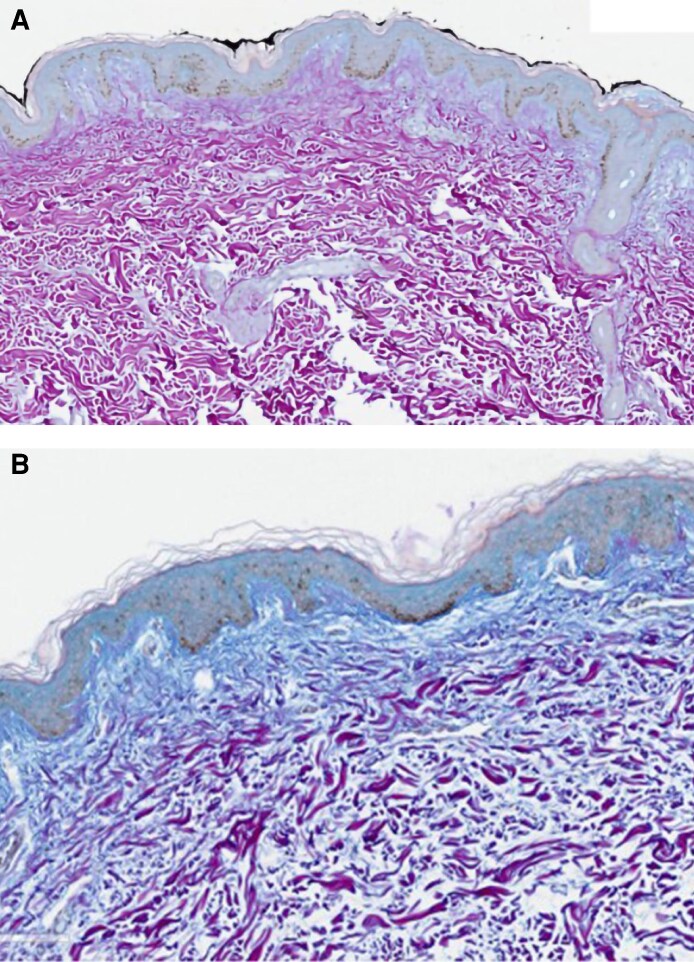
Histological changes—Herovici stain used to demonstrate early mucopolysaccharide formation, indicating early collagen fiber development. This appears as a shift from magenta color (mature older collagen) to blue color (early collagen fiber formation), clearly shown here at (A) baseline vs (B) 12 weeks. Neocollagenesis with improved extracellular matrix changes in B at 12 weeks compared with baseline in A.

The findings across these studies relate to how subcutaneous fibrous banding affects faster resolution (Figures 1, 2). PROMs showed earlier recovery, less discomfort, and less induration on the treated sides.^3^ Ultrasound revealed fewer hyperechoic fibrous strands in the subcutaneous tissue (Figure 3), and histology showed ECM remodeling (Figure 4).

### External Scarring

**Figure 5.**
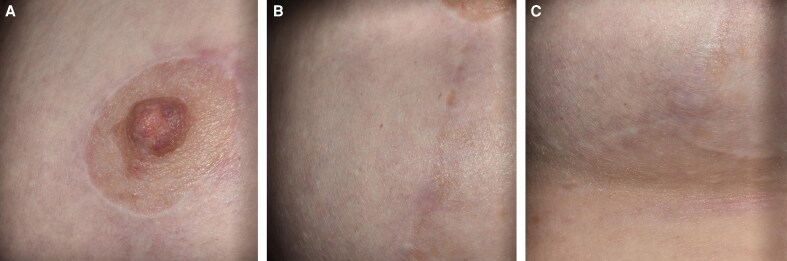
(A, B, C) Typical scars at 6 months after breast surgery with R&R use; patient is a 52-year-old female who underwent a breast reduction procedure—periareolar area, vertical, and inframammary incisions all appear flat, with a white, nonreactive edge, representing a common outcome for what is considered good scarring.

R&R improved incision line appearance with early softening, decreased erythema, and overall better scar quality (Figure 5).

## DISCUSSION

**Figure 6.**
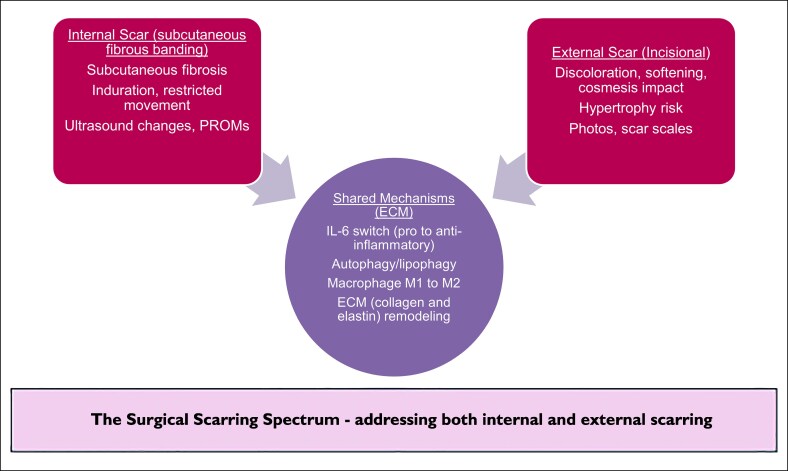
Diagrammatic representation of the relationship between internal (fibrous banding) and external (incisional) scarring. Both are connected through common mechanistic pathways involving ECM remodeling (collagen and elastin), adipocytolysis, IL-6 regulation, autophagy, and macrophage polarization. R&R accelerates healing across this spectrum. ECM, extracellular matrix; IL-6, interleukin-6; PROM, patient-reported outcome measure.

The spectrum of surgical scarring described in this paper includes both deeper fibrous bands and superficial incisional scars. Both types are driven by ECM dysregulation and reformation. They are influenced and regulated by R&R modulation of inflammation, specifically the orchestration of processes by IL-6, autophagy, and macrophage polarization (Figure 6).^7^ The clinical data reflect these molecular changes, showing improvements in scar appearance, reduced discomfort, and faster recovery after surgery.

**Figure 7.**
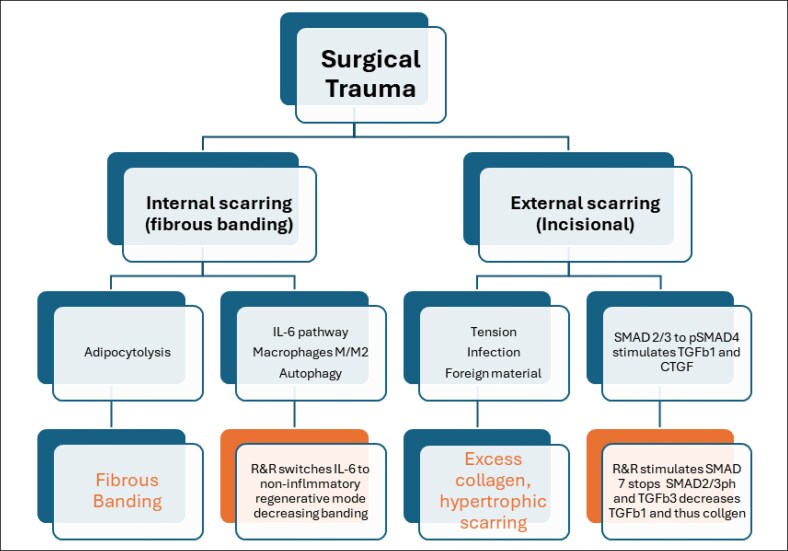
Parallel mechanistic pathways demonstrate how surgical trauma produces both internal and external scarring. Internal pathway: adipocytolysis and IL-6/macrophage signaling resolve through autophagy and R&R-mediated regenerative remodeling to reduce fibrous banding. External pathway: infection/foreign material and tension trigger mechanoreceptor-driven SMAD2/3→SMAD4 activation and TGF-β1/CTGF upregulation, counteracted by R&R-induced SMAD7 and TGF-β3. Both converge toward normalized ECM regeneration and balanced scar outcomes. CTGF, connective tissue growth factor; ECM, extracellular matrix; IL-6, interleukin-6; SMAD, mothers against decapentaplegic homolog; TGF-β, transforming growth factor beta.

In addition to the shared mechanisms described above, there are esoteric pathways involved in external scarring that are also managed by R&R. Thus, external scarring can be affected by tension, infection, foreign materials. This initiates signaling through mechanoreceptors with stimulation of SMAD pathway, phosphorylation of SMAD2/3 to SMAD4 facilitating entry to the fibroblast cell and nuclear transcription resulting the stimulation of TGF-β1 and connective tissue growth factor (CTGF).^8^ R&R increases SMAD7 and TGF-β3, which inhibit SMAD2/3→SMAD4 phosphorylation and counteracts TGF-β1/CTGF, normalizing collagen maturation (Figure 7). Thus, surgical trauma triggers 2 parallel—but mechanistically connected—scarring pathways:

- Internal scarring (subcutaneous fibrous banding) where tissue injury causes adipocytolysis, resulting in the release of lipid droplets, increased IL-6, and prolonged inflammation. R&R promotes autophagy/lipophagy and M1–M2 macrophage polarization, helping switch the “IL-6 switch” from inflammation to regeneration. The downstream effect is turning off inflammation, switching on regeneration, ECM remodeling, and a reduction in fibrous banding.
- External scarring (incisional) which can be triggered by infection, foreign material, and mechanical forces (tension) that activate mechanoreceptors. This initiates phosphorylation of SMAD2/3, formation of SMAD4 complexes, and upregulation of TGF-β1 and CTGF leading to excess collagen deposition and scar hypertrophy. Tape support (mechanical off-loading) and R&R act both upstream and downstream: tape reduces mechanotransduction; R&R increases SMAD7 and TGF-β3, which inhibit SMAD2/3→SMAD4 signaling and counteract TGF-β1/CTGF, guiding controlled collagen maturation and resulting in a flatter, lighter incision line.

### Unified Concept

Internal and external scarring are points on a single spectrum influenced by inflammation control, ECM clearance, and collagen/elastin remodeling. R&R targets both through separate mechanisms (IL-6/autophagy/macrophages internally; SMAD7/TGF-β3 antagonism of SMAD/TGF-β1/CTGF externally), ultimately leading to regenerative ECM remodeling. Tape support and hydration enhance these external effects by reducing the initial mechanical signal. Internal fibrous banding has rarely been reported as an outcome despite affecting comfort and mobility. The evidence from PROMs, investigator reports, ultrasound, and histology has identified this outcome as a measurable factor worth analysis as a surgical endpoint.

Naturally, external scarring remains an important outcome for patients, with ongoing focus on discoloration, softening, and flattening of scars as desired results.

The results from the trials analyzed above support the idea that a topical preparation can influence surgical outcomes. The parameters examined include common measures, such as ecchymoses, edema, and external scarring. However, evaluating internal scarring through fibrous banding introduces a new measurable outcome that affects patient comfort and recovery. Using a single topical agent for preconditioning, as well as immediate and extended postsurgery use, has proven to be beneficial.

### Limitations and Future Considerations

Scar-specific endpoints were not a primary focus in previous studies, and prospective trials focused on external scarring are needed and planned. Further analysis of variations in skin types, nuances of different body contouring surgical procedures, and the direct correlation between external and internal scarring are areas that would provide additional insight into the scarring spectrum Additionally, it may be considered that the natural wound healing process is being interfered with and could alter the normal wound healing mechanism. However, our gene expression studies demonstrate a resolution of inflammation and a shift in macrophage polarization from M1 to M2 macrophages, indicating a transition from inflammation to regeneration.^6^ This is reflected in the histological changes observed in the ECM. Therefore, we believe that we are accelerating the resolution of inflammation and thereby promoting a more efficient start to regenerative processes, rather than delaying wound healing.

Aesthetic outcomes and external scar appearance have traditionally been used to measure surgical recovery and success. However, recovery time, patient discomfort, and regained mobility are crucial aspects that help patients return to their normal lives more quickly and with less disruption. This process can be affected by internal scarring or subcutaneous fibrous banding, a condition that has not yet been a focus as a measurable outcome. Fibrous banding and external scarring are linked to ECM disruption and molecular changes directly tied to inflammatory pathways. These can be influenced by modulating IL-6 control, which can trigger a regenerative response and reverse fibrosis across the spectrum of surgical scarring.

## CONCLUSIONS

Multiple trials have been analyzed to assess the effect of a topical formulation on scarring. R&R influences the spectrum of surgical scarring, affecting both internal and external scars. The unified mechanistic theory of scar modulation broadens traditional views of postoperative healing, emphasizing internal fibrous banding as a measurable outcome and positioning a topical formulation as a credible additional tool in perisurgical care. R&R as a topical formulation has proven effective in impacting internal and external scarring. By understanding and treating these issues, better patient outcomes and satisfaction are expected, with shorter recovery times.
